# Life as we know it

**DOI:** 10.1098/rsif.2013.0475

**Published:** 2013-09-06

**Authors:** Karl Friston

**Affiliations:** The Wellcome Trust Centre for Neuroimaging, Institute of Neurology, Queen Square, London WC1N 3BG, UK

**Keywords:** autopoiesis, self-organization, active inference, free energy, ergodicity, random attractor

## Abstract

This paper presents a heuristic proof (and simulations of a primordial soup) suggesting that life—or biological self-organization—is an inevitable and emergent property of any (ergodic) random dynamical system that possesses a Markov blanket. This conclusion is based on the following arguments: if the coupling among an ensemble of dynamical systems is mediated by short-range forces, then the states of remote systems must be conditionally independent. These independencies induce a Markov blanket that separates internal and external states in a statistical sense. The existence of a Markov blanket means that internal states will appear to minimize a free energy functional of the states of their Markov blanket. Crucially, this is the same quantity that is optimized in Bayesian inference. Therefore, the internal states (and their blanket) will appear to engage in active Bayesian inference. In other words, they will appear to model—and act on—their world to preserve their functional and structural integrity, leading to homoeostasis and a simple form of autopoiesis.

## 1. Introduction

How can the events in space and time which take place within the spatial boundary of a living organism be accounted for by physics and chemistry?

Erwin Schrödinger [1, p. 2]

The emergence of life—or biological self-organization—is an intriguing issue that has been addressed in many guises in the biological and physical sciences [1–5]. This paper suggests that biological self-organization is not as remarkable as one might think—and is (almost) inevitable, given local interactions between the states of coupled dynamical systems. In brief, the events that ‘take place within the spatial boundary of a living organism’ [1] may arise from the very existence of a boundary or blanket, which itself is inevitable in a physically lawful world.

The treatment offered in this paper is rather abstract and restricts itself to some basic observations about how coupled dynamical systems organize themselves over time. We will only consider behaviour over the timescale of the dynamics themselves—and try to interpret this behaviour in relation to the sorts of processes that unfold over seconds to hours, e.g. cellular processes. Clearly, a full account of the emergence of life would have to address multiple (evolutionary, developmental and functional) timescales and the emergence of DNA, ribosomes and the complex cellular networks common to most forms of life. This paper focuses on a simple but fundamental aspect of self-organization—using abstract representations of dynamical processes—that may provide a metaphor for behaviour with different timescales and biological substrates.

Most treatments of self-organization in theoretical biology have addressed the peculiar resistance of biological systems to the dispersive effects of fluctuations in their environment by appealing to statistical thermodynamics and information theory [1,3,5–10]. Recent formulations try to explain adaptive behaviour in terms of minimizing an upper (free energy) bound on the *surprise* (negative log-likelihood) of sensory samples [11,12]. This minimization usefully connects the imperative for biological systems to maintain their sensory states within physiological bounds, with an intuitive understanding of adaptive behaviour in terms of active inference about the causes of those states [13].

Under ergodic assumptions, the long-term average of surprise is entropy. This means that minimizing free energy—through selectively sampling sensory input—places an upper bound on the entropy or dispersion of sensory states. This enables biological systems to resist the second law of thermodynamics—or more exactly the fluctuation theorem that applies to open systems far from equilibrium [14,15]. However, because negative surprise is also *Bayesian model evidence*, systems that minimize free energy also maximize a lower bound on the evidence for an implicit model of how their sensory samples were generated. In statistics and machine learning, this is known as *approximate Bayesian inference* and provides a normative theory for the Bayesian brain hypothesis [16–20]. In short, biological systems act on the world to place an upper bound on the dispersion of their sensed states, while using those sensations to infer external states of the world. This inference makes the free energy bound a better approximation to the surprise that action is trying to minimize [21]. The resulting active inference is closely related to formulations in embodied cognition and artificial intelligence; for example, the use of predictive information [22–24] and earlier homeokinetic formulations [25].

The ensuing (variational) free energy principle has been applied widely in neurobiology and has been generalized to other biological systems at a more theoretical level [11]. The motivation for minimizing free energy has hitherto used the following sort of argument: systems that do not minimize free energy cannot exist, because the entropy of their sensory states would not be bounded and would increase indefinitely—by the fluctuation theorem [15]. Therefore, biological systems must minimize free energy. This paper resolves the somewhat tautological aspect of this argument by turning it around to suggest: any system that exists will appear to minimize free energy and therefore engage in active inference. Furthermore, this apparently inferential or mindful behaviour is (almost) inevitable. This may sound like a rather definitive assertion but is surprisingly easy to verify. In what follows, we will consider a heuristic proof based on random dynamical systems and then see that biological self-organization emerges naturally, using a synthetic primordial soup. This proof of principle rests on four attributes of—or tests for—self-organization that may themselves have interesting implications.

## 2. Heuristic proof

We start with the following lemma: *any ergodic random dynamical system that possesses a Markov blanket will appear to actively maintain its structural and dynamical integrity*. We will associate this behaviour with the self-organization of living organisms. There are two key concepts here—*ergodicity* and a *Markov blanket*. Here, ergodicity means that the time average of any measurable function of the system converges (almost surely) over a sufficient amount of time [26,27]. This means that one can interpret the average amount of time a state is occupied as the probability of the system being in that state when observed at random. We will refer to this probability measure as the *ergodic density*.

A Markov blanket is a set of states that separates two other sets in a statistical sense. The term Markov blanket was introduced in the context of Bayesian networks or graphs [28] and refers to the children of a set (the set of states that are influenced), its parents (the set of states that influence it) and the parents of its children. The notion of influence or dependency is central to a Markov blanket and its existence implies that any state is—or is not—coupled to another. For example, the system could comprise an ensemble of subsystems, each occupying its own position in a Euclidean space. If the coupling among subsystems is mediated by short-range forces, then distant subsystems cannot influence each other. The existence of a Markov blanket implies that its states (e.g. motion in Euclidean space) do not affect their coupling or independence. In other words, the interdependencies among states comprising the Markov blanket change slowly with respect to the states *per se*. For example, the surface of a cell may constitute a Markov blanket separating intracellular and extracellular states. On the other hand, a candle flame cannot possess a Markov blanket, because any pattern of molecular interactions is destroyed almost instantaneously by the flux of gas molecules from its surface.

**Figure 1.**
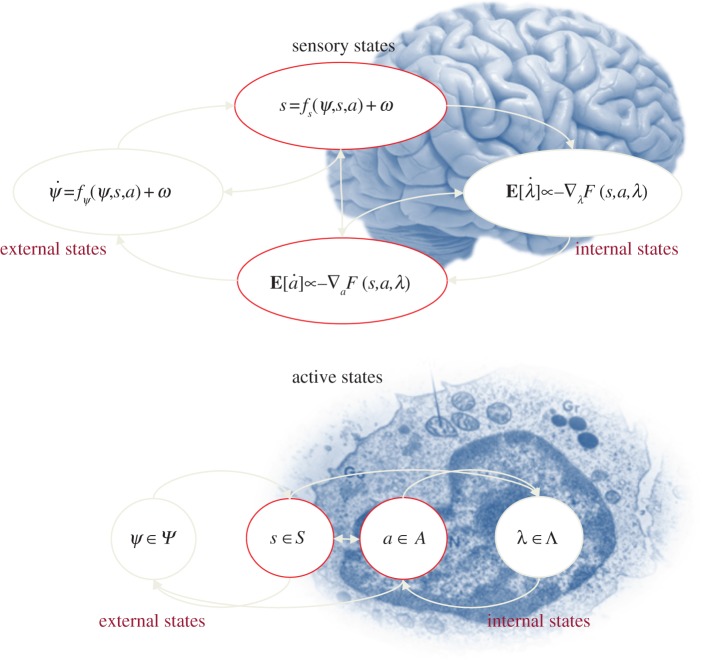
Markov blankets and the free energy principle. These schematics illustrate the partition of states into internal states and hidden or external states that are separated by a Markov blanket—comprising sensory and active states. The upper panel shows this partition as it would be applied to action and perception in the brain; where—in accord with the free energy principle—active and internal states minimize a free energy functional of sensory states. The ensuing self-organization of internal states then corresponds to perception, while action couples brain states back to external states. The lower panel shows exactly the same dependencies but rearranged so that the internal states can the associated with the intracellular states of a cell, while the sensory states become the surface states or cell membrane overlying active states (e.g. the actin filaments of the cytoskeleton). See table 1 for a definition of variables.

The existence of a Markov blanket induces a partition of states into *internal* states and *external* states that are hidden (insulated) from the internal (insular) states by the Markov blanket. In other words, the external states can only be seen vicariously by the internal states, through the Markov blanket. Furthermore, the Markov blanket can itself be partitioned into two sets that are, and are not, children of external states. We will refer to these as a surface or *sensory states* and *active states*, respectively. Put simply, the existence of a Markov blanket *S* × *A* implies a partition of states into external, sensory, active and internal states: 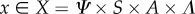. External states cause sensory states that influence—but are not influenced by—internal states, while internal states cause active states that influence—but are not influenced by—external states (table 1). Crucially, the dependencies induced by Markov blankets create a circular causality that is reminiscent of the action–perception cycle (figure 1). The circular causality here means that external states cause changes in internal states, via sensory states, while the internal states couple back to the external states through active states—such that internal and external states cause each other in a reciprocal fashion. This circular causality may be a fundamental and ubiquitous causal architecture for self-organization.

**Table 1.** Definitions of the tuple 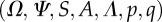 underlying active inference*.*

*a sample space Ω* or non-empty set from which random fluctuations or outcomes *ω* ∈ *Ω* are drawn
*external states* 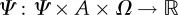 states of the world that cause sensory states and depend on action
*sensory states* 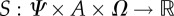 the agent's sensations that constitute a probabilistic mapping from action and external states
*action states* 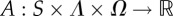 an agent's action that depends on its sensory and internal states
*internal states* 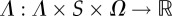 the states of the agent that cause action and depend on sensory states
*ergodic density* 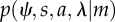 a probability density function over external *ψ* ∈ *Ψ*, sensory *s ∈ S*, active *a ∈ A* and internal states *λ* ∈ *Λ* for a system denoted by *m*
*variational density q*(*ψ*|*λ*) an arbitrary probability density function over external states that is parametrized by internal states

Equipped with this partition, we can now consider the behaviour of any random dynamical system *m* described by some stochastic differential equations:2.1
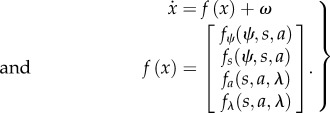


Here, *f*(*x*) is the flow of system states that is subject to random fluctuations denoted by *ω*. The second equality formalizes the dependencies implied by the Markov blanket. Because the system is ergodic it will, after a sufficient amount of time, converge to an invariant set of states called a *pullback* or *random global attractor*. The attractor is random because it itself is a random set [29,30]. The associated ergodic density *p*(*x|m*) is the solution to the Fokker–Planck equation (a.k.a. the Kolmogorov forward equation) [31] describing the evolution of the probability density over states2.2
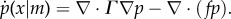


Here, the diffusion tensor *Γ* is the half the covariance (amplitude) of the random fluctuations. Equation (2.2) shows that the ergodic density depends upon flow, which can always be expressed in terms of curl and divergence-free components. This is the Helmholtz decomposition (a.k.a. the fundamental theorem of vector calculus) and can be formulated in terms of an antisymmetric matrix *R*(*x*) = *−R*(*x*)^T^ and a scalar potential *G*(*x*) we will call Gibbs energy [32],2.3
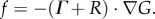


Using this standard form [33], it is straightforward to show that *p*(*x|m*) = exp(*−G*(*x*)) is the equilibrium solution to the Fokker–Planck equation [12]:2.4
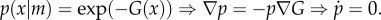


This means that we can express the flow in terms of the ergodic density2.5
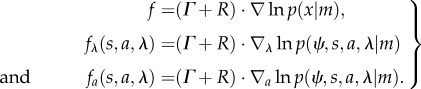


Although we have just followed a sequence of standard results, there is something quite remarkable and curious about this flow: the flow of internal and active states is essentially a (circuitous) gradient ascent on the (log) ergodic density. The gradient ascent is circuitous because it contains divergence-free (solenoidal) components that circulate on the isocontours of the ergodic density—like walking up a winding mountain path. This ascent will make it look as if internal (and active) states are flowing towards regions of state space that are most frequently occupied *despite the fact their flow is not a function of external states*. In other words, their flow does not depend upon external states (see the right-hand side equation (2.5)) and yet it ascends gradients that depend on the external states (see the right-hand side of equation (2.5)). In short, the internal and active states behave as if they know where they are in the space of external states—states that are hidden behind the Markov blanket.

We can finesse this apparent paradox by noting that the flow is the expected motion through any point averaged over time. By the ergodic theorem, this is also the flow averaged over the external states, which does not depend on the external state at any particular time: more formally, for any point *v∈V* = *S* × *A* × *Λ* in the space of the internal states and their Markov blanket, equations (2.1) and (2.5) tell us that flow through this point is the average flow under the posterior density over the external states:


2.6
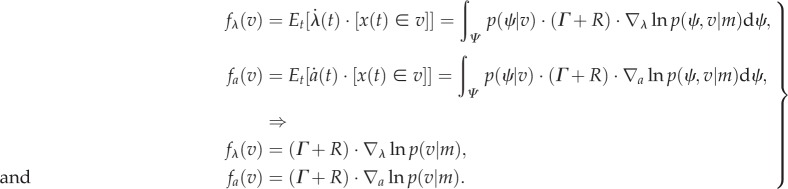


The Iverson bracket [*x*(*t*) ∈ *v*] returns a value of one when the trajectory passes through the point in question and zero otherwise—and the first expectation is taken over time. Here, we have used the fact that the integral of a derivative of a density is the derivative of its integral—and both are zero.

Equation (2.6) is quite revealing—it shows that the flow of internal and active states performs a circuitous gradient ascent on the *marginal* ergodic density over internal states and their Markov blanket. Crucially, this marginal density depends on the posterior density over external states. This means that the internal states will appear to respond to sensory fluctuations based on posterior beliefs about underlying fluctuations in external states. We can formalize this notion by associating these beliefs with a probability density over external states *q*(*ψ*|*λ*) that is encoded (parametrized) by internal states.

**Lemma 2.1**

**Free energy**. *For any Gibbs energy G*(*ψ*, *s*, *a*, *λ*) = −ln *p*(*ψ*, *s*, *a*, *λ*), *there is a free energy F*(*s, a, λ*) *that describes the flow of internal and active states*:2.7
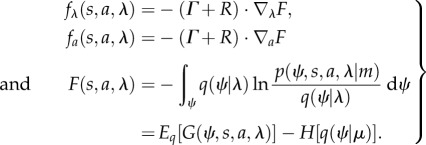

*Here*, *free energy is a functional of an arbitrary* (*variational*) *density q*(*ψ*|*λ*) *that is parametrized by internal states. The last equality just shows that free energy can be expressed as the expected Gibbs energy minus the entropy of the variational density*.

**Proof.**

Using Bayes rule, we can rearrange the expression for free energy in terms of a Kullback*–*Leibler divergence [34]:2.8
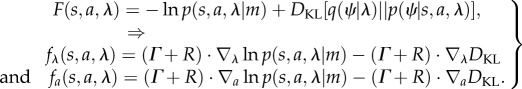
However, equation (2.6) requires the gradients of the divergence to be zero, which means the divergence must be minimized with respect to internal states. This means that the variational and posterior densities must be equal:
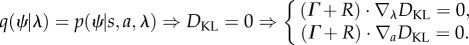

In other words, the flow of internal and active states minimizes free energy, rendering the variational density equivalent to the posterior density over external states.

**Remarks 2.2.** Put simply, this proof says that if one interprets internal states as parametrizing a variational density encoding Bayesian beliefs about external states, then the dynamics of internal and active states can be described as a gradient descent on a variational free energy function of internal states and their Markov blanket. Variational free energy was introduced by Feynman [35] to solve difficult integration problems in path integral formulations of quantum physics. This is also the free energy bound that is used extensively in *approximate Bayesian inference* (e.g. variational Bayes) [34,36,37]. The expression for free energy in equation (2.8) discloses its Bayesian interpretation: the first term is the negative log evidence or *marginal likelihood* of the internal states and their Markov blanket. The second term is a *relative entropy* or Kullback–Leibler divergence [38] between the variational density and the posterior density over external states. Because (by Gibbs inequality) this divergence cannot be less than zero, the internal flow will appear to have minimized the divergence between the variational and posterior density. In other words, the internal states will appear to have solved the problem of Bayesian inference by encoding posterior beliefs about hidden (external) states, under a generative model provided by the Gibbs energy. This is known as *approximate* Bayesian inference—with *exact* Bayesian inference when the forms of the variational and posterior densities are identical. In short, the internal states will appear to engage in some form of Bayesian inference: but what about action?

Because the divergence in equation (2.8) can never be less than zero, free energy is an upper bound on the negative log evidence. Now, because the system is ergodic we have2.9
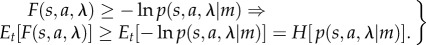


This means that action will (on average) appear to minimize free energy and thereby place an upper bound on the entropy of the internal states and their Markov blanket. If we associate these states *v* = {*s*, *a*, *λ*} with biological systems, then action places an upper bound on their dispersion (entropy) and will appear to conserve their structural and dynamical integrity. Together with the Bayesian modelling perspective, this is exactly consistent with the good regulator theorem (every good regulator is a model of its environment) and related treatments of self-organization [2,5,12,39,40]. Furthermore, we have shown elsewhere [11,41] that free energy minimization is consistent with information-theoretic formulations of sensory processing and behaviour [23,42,43]. Equation (2.7) also shows that minimizing free energy entails maximizing the entropy of the variational density (the final term in the last equality)—in accord with the maximum entropy principle [44]. Finally, because we have cast this treatment in terms of random dynamical systems, there is an easy connection to dynamical formulations that predominate in the neurosciences [40,45–47].

The above arguments can be summarized with the following attributes of biological self-organization:

— *biological systems are ergodic* [26]: in the sense that the average of any measure of their states converges over a sufficient period of time. This includes the occupancy of state space and guarantees the existence of an invariant ergodic density over functional and structural states;
— *they are equipped with a Markov blanket* [28]: the existence of a Markov blanket necessarily implies a partition of states into internal states, their Markov blanket (sensory and active states) and external or hidden states. Internal states and their Markov blanket (*biological states*) constitute a biological system that responds to hidden states in the environment;
— *they exhibit active inference* [11]: the partition of states implied by the Markov blanket endows internal states with the apparent capacity to represent hidden states probabilistically, so that they appear to infer the hidden causes of their sensory states (by minimizing a free energy bound on log Bayesian evidence). By the circular causality induced by the Markov blanket, sensory states depend on active states, rendering inference active or embodied; and
— *they are autopoietic* [4]: because active states change—but are not changed by—hidden states (figure 1), they will appear to place an upper (free energy) bound on the dispersion (entropy) of biological states. This homoeostasis is informed by internal states, which means that active states will appear to maintain the structural and functional integrity of biological states.

When expressed like this, these criteria appear perfectly sensible but are they useful in the setting of real biophysical systems? The premise of this paper is that these criteria apply to (almost) all ergodic systems encountered in the real world. The argument here is that biological behaviour rests on the existence of a Markov blanket—and that a Markov blanket is (almost) inevitable in coupled dynamical systems with short-range interactions. In other words, if the coupling between dynamical systems can be neglected—when they are separated by large distances—the intervening systems will necessarily form a Markov blanket. For example, if we consider short-range electrochemical and nuclear forces, then a cell membrane forms a Markov blanket for internal intracellular states (figure 1). If this argument is correct, then it should be possible to show the emergence of biological self-organization in any arbitrary ensemble of coupled subsystems with short-range interactions. The final section uses simulations to provide a proof of principle, using the four criteria above to identify and verify the emergence of lifelike behaviour.

## 3. Proof of principle

In this section, we simulate a primordial soup to illustrate the emergence of biological self-organization. This soup comprises an ensemble of dynamical subsystems—each with its own structural and functional states—that are coupled through short-range interactions. These simulations are similar to (hundreds of) simulations used to characterize pattern formation in dissipative systems; for example, Turing instabilities [48]: the theory of dissipative structures considers far-from-equilibrium systems, such as turbulence and convection in fluid dynamics (e.g. Bénard cells), percolation and reaction–diffusion systems such as the Belousov–Zhabotinsky reaction [49]. Self-assembly is another important example from chemistry that has biological connotations (e.g. for pre-biotic formation of proteins). The simulations here are distinguished by solving stochastic differential equations for both structural and functional states. In other words, we consider states from classical mechanics that determine physical motion—and functional states that could describe electrochemical states. Importantly, the functional states of any system affect the functional and structural states of another. The agenda here is not to explore the repertoire of patterns and self-organization these ensembles exhibit—but rather take an arbitrary example and show that, buried within it, there is a clear and discernible anatomy that satisfies the criteria for life.

### 3.1. The primordial soup

To simulate a primordial soup, we use an ensemble of elemental subsystems with (heuristically speaking) Newtonian and electrochemical dynamics 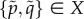:3.1
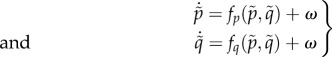


Here, 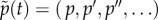 are generalized coordinates of motion describing position, velocity, acceleration—and so on—of the subsystems, while 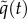 correspond to electrochemical states (such as concentrations or electromagnetic states). One can think of these generalized states as describing the physical and electrochemical state of large macromolecules. Crucially, these states are coupled within and between the subsystems comprising an ensemble. The electrochemical dynamics were chosen to have a Lorenz attractor: for the *i*th system with its own rate parameter *κ*^(*i*)^:3.2
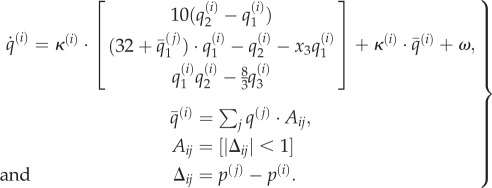
Changes in electrochemical states are coupled through the local average 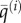
of the states of subsystems that lie within a distance of one. This means that *A* can be regarded as an (unweighted) adjacency matrix that encodes the dependencies among the functional (electrochemical) states of the ensemble. The local average enters the equations of motion both linearly and nonlinearly to provide an opportunity for generalized synchronization [50]. The nonlinear coupling effectively renders the Rayleigh parameter of the flow 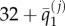 state-dependent.

The Lorenz form for these dynamics is a somewhat arbitrary choice but provides a ubiquitous model of electrodynamics, lasers and chemical reactions [51]. The rate parameter 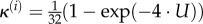 was specific to each subsystem, where *U* ∈ (0, 1) was selected from a uniform distribution. This introduces heterogeneity in the rate of electrochemical dynamics, with a large number of fast subsystems—with a rate constant of nearly one—and a small number of slower subsystems. To augment this heterogeneity, we randomly selected a third of the subsystems and prevented them from (electrochemically) influencing others, by setting the appropriate column of the adjacency matrix to zero. We refer to these as functionally *closed* systems.

In a similar way, the classical (Newtonian) motion of each subsystem depends upon the functional status of its neighbours:3.3
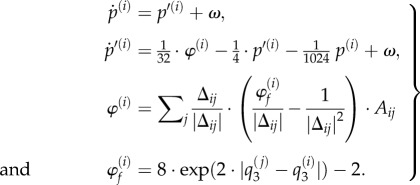


This motion rests on forces *φ*^(*i*)^ exerted by other subsystems that comprise a strong repulsive force (with an inverse square law) and a weaker attractive force that depends on their electrochemical states. This force was chosen so that systems with coherent (third) states are attracted to each other but repel otherwise. The remaining two terms in the expression for acceleration (second equality) model viscosity that depends upon velocity and an exogenous force that attracts all locations to the origin—as if they were moving in a simple (quadratic) potential energy well. This ensures the synthetic soup falls to the bottom of the well and enables local interactions.

Note that the ensemble system is dissipative at two levels: first, the classical motion includes dissipative friction or viscosity. Second, the functional dynamics are dissipative in the sense that they are not divergence-free. We will now assess the criteria for biological self-organization within this coupled random dynamical ensemble.

### 3.2. Ergodicity

In the examples used below, 128 subsystems were integrated using Euler's (forward) method with step sizes of 1/512 s and initial conditions sampled from the normal distribution. Random fluctuations were sampled from the unit normal distribution. By adjusting the parameters in the above equations of motion, one can produce a repertoire of plausible and interesting behaviours (the code for these simulations and the figures in this paper are available as part of the SPM academic freeware). These behaviours range from gas-like behaviour (where subsystems occasionally get close enough to interact) to a cauldron of activity, when subsystems are forced together at the bottom of the potential well. In this regime, subsystems get sufficiently close for the inverse square law to blow them apart—reminiscent of subatomic particle collisions in nuclear physics. With particular parameter values, these sporadic and critical events can render the dynamics non-ergodic, with unpredictable high amplitude fluctuations that do not settle down. In other regimes, a more crystalline structure emerges with muted interactions and low structural (configurational) entropy.

**Figure 2.**
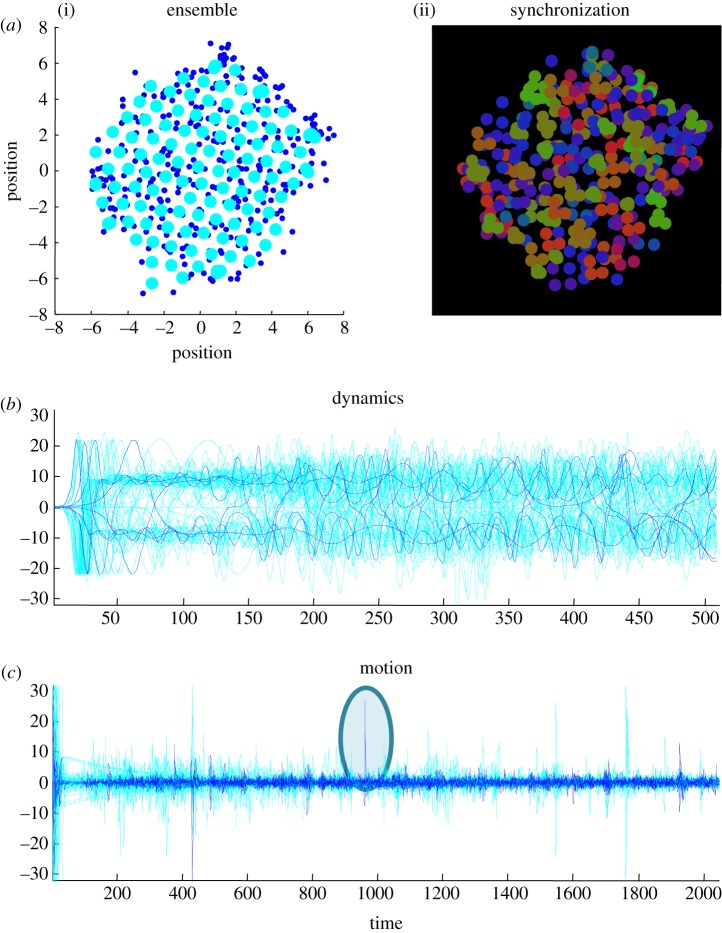
Ensemble dynamics. (*a*) The position of (128) subsystems comprising an ensemble after 2048 s. *a*(i) The dynamical status (three blue dots per subsystem) of each subsystem centred on its location (larger cyan dots). *a*(ii) The same information, where the relative values of the three dynamical states of each subsystem are colour-coded (using a softmax function of the three functional states and a RGB mapping). This illustrates the synchronization of dynamical states within each subsystem and the dispersion of the phases of the Lorenzian dynamics over subsystems. (*b*,*c*) The evolution of functional  and structural states as a function of time, respectively. The (electrochemical) dynamics of the internal (blue) and external (cyan) states are shown for the 512 s. One can see initial (chaotic) transients that resolve fairly quickly, with itinerant behaviour as they approach their attracting set. (*c*) The position of internal (blue) and external (cyan) subsystems over the entire simulation period illustrate critical events (circled) that occur every few hundred seconds, especially at the beginning of the simulation. These events generally reflect a pair of particles (subsystems) being expelled from the ensemble to the periphery, when they become sufficiently close to engage short-range repulsive forces. These simulations integrated the stochastic differential equations in the main text using a forward Euler method with 1/512 s time steps and random fluctuations of unit variance.

However, for most values of the parameters, ergodic behaviour emerges as the ensemble approaches its random global attractor (usually after about 1000 s): generally, subsystems repel each other initially (much like illustrations of the big bang) and then fall back towards the centre, finding each other as they coalesce. Local interactions then mediate a reorganization, in which subsystems are passed around (sometimes to the periphery) until neighbours gently jostle with each other. In terms of the dynamics, transient synchronization can be seen as waves of dynamical bursting (due to the nonlinear coupling in equation (3.2)). In brief, the motion and electrochemical dynamics look very much like a restless soup (not unlike solar flares on the surface of the sun, figure 2)—but does it have any self-organization beyond this?

### 3.3. The Markov blanket

Because the structural and functional dependencies share the same adjacency matrix—which depends upon position—one can use the adjacency matrix to identify the principal Markov blanket by appealing to spectral graph theory: the Markov blanket of any subset of states encoded by a binary vector with elements *χ_i_ ∈* {0, 1} is given by [*B · χ*] ∈ {0, 1}, where the Markov blanket matrix *B* = *A* + *A*^T^ + *A*^T^*A* encodes children, parents and parents of children. This follows because the *i*th column of the adjacency matrix encodes the directed connections from the *i*th state to all its children. The principal eigenvector of the (symmetric) Markov blanket matrix will—by the Perron–Frobenius theorem—contain positive values. These values reflect the degree to which each state belongs to the cluster that is most interconnected (cf., spectral clustering). In what follows, the internal states were defined as belonging to subsystems with the *k* = 8 largest values. Having defined the internal states, the Markov blanket can be recovered from the Markov blanket matrix using [*B · χ*] and divided into sensory and active states—depending upon whether they are influenced by the hidden states or not.

**Figure 3.**
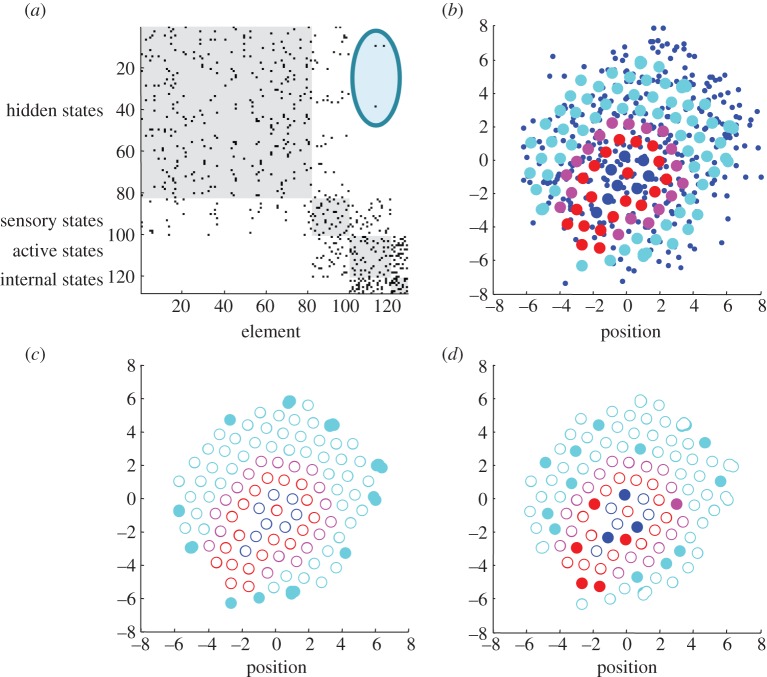
Emergence of the Markov blanket. (*a*) The adjacency matrix that indicates a conditional dependency (spatial proximity) on at least one occasion over the last 256 s of the simulation. The adjacency matrix has been reordered to show the partition of hidden (cyan), sensory (magenta), active (red) and internal (blue) subsystems, whose positions are shown in (*b*)—using the same format as in the previous figure. Note the absence of direct connections (edges) between external or hidden and internal subsystem states. The circled area illustrates coupling between active and hidden states that are not reciprocated (there are no edges between hidden and active states). The spatial self-organization in the upper left panel is self evident; where the internal states have arranged themselves in a small loop structure with a little cilium, protected by the active states that support the surface or sensory states. When viewed as a movie, the entire ensemble pulsates in a chaotic but structured fashion, with the most marked motion in the periphery. (*c*,*d*) Highlights those subsystems that cannot influence others (closed subsystems (*c*)) and those that have slower dynamics (slow subsystems (*d*)). The remarkable thing here is that all the closed subsystems have been rusticated to the periphery—where they provide a locus for vigorous dynamics and motion. Contrast this with the deployment of slow subsystems that are found throughout the hidden, sensory, active and internal partition.

Given the internal states and their Markov blanket, we can now follow their assembly and visualize any structural or functional characteristics. Figure 3 shows the adjacency matrix used to identify the Markov blanket. This adjacency matrix has non-zero entries if two subsystems were coupled over the last 256 s of a 2048 s simulation. In other words, it accommodates the fact that the adjacency matrix is itself an ergodic process—due to the random fluctuations. Figure 3*b* shows the location of subsystems with internal states (blue) and their Markov blanket—in terms of sensory (magenta) and active (red) locations. A clear structure can be seen here, where the internal subsystems are (unsurprisingly) close together and enshrouded by the Markov blanket. Interestingly, the active subsystems support the sensory subsystems that are exposed to hidden environmental states. This is reminiscent of a biological cell with a cytoskeleton that supports some sensory epithelia or receptors within its membrane.

Figure 3*c* highlights functionally closed subsystems (filled circles) that have been rusticated to the periphery of the system. Recall that these subsystems cannot influence or engage other subsystems and are therefore expelled to the outer limits of the soup. Heuristically, they cannot invade the system and establish a reciprocal and synchronous exchange with other subsystems. Interestingly, no simulation ever produced a functionally closed internal state. Figure 3*d* shows the slow subsystems that are distributed between internal and external states—which may say something interesting about the generalized synchrony that underlies self-organization.

### 3.4. Active inference

If the internal states encode a probability density over the hidden or external states, then it should be possible to predict external states from internal states. In other words, if internal events represent external events, they should exhibit a significant statistical dependency. To establish this dependency, we examined the functional (electrochemical) status of internal subsystems to see whether they could predict structural events (movement) in the external milieu. This is not unlike the approach taken in brain mapping that searches for statistical dependencies between, say, motion in the visual field and neuronal activity [52].

**Figure 4.**
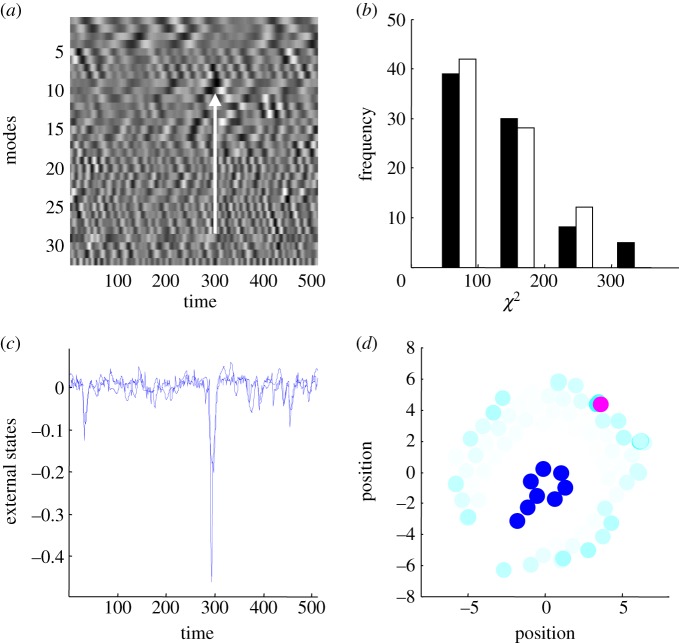
Self-organized perception. This figure illustrates the Bayesian perspective on self-organized dynamics. (*a*) The first (principal) 32 eigenvariates of the internal (functional) states as a function of time over the last 512 s of the simulations reported in the previous figures. These eigenvariates were obtained by a singular value decomposition of the timeseries over all internal functional states (lagged between plus and minus 16 s). These represent a summary of internal dynamics that are distributed over internal subsystems. The eigenvariates were then used to predict the (two-dimensional) motion of each external subsystem using a standard canonical variates analysis. The (classical) significance of this prediction was assessed using Wilks' lambda (following a standard transformation to the χ^2^ statistic). The actual (dotted line) and predicted (solid line) position for the most significant external subsystem is shown in (*c*)—in terms of canonical variates (best linear mixture of position in two dimensions). The agreement is self-evident and is largely subtended by negative excursions, notably at 300 s. The fluctuations in internal states are visible in (*a*) and provide a linear mixture that correlates with the external fluctuation (highlighted with a white arrow). The location of the external subsystem that was best predicted is shown by the magenta circle on (*d*). Remarkably, this is the subsystem that is the furthest away from the internal states and is one of the subsystems that participates in the exchanges a closed subsystem in the previous figure. (*c*) Also shows the significance with which the motion of the remaining external states could be predicted (with the intensity of the cyan being proportional to the χ^2^ statistic above). Interestingly, the motion that is predicted with the greatest significance is restricted to the periphery of the ensemble, where the external subsystems have the greatest latitude for movement. To ensure this inferential coupling was not a chance phenomenon, we repeated the analysis after flipping the external states in time. This destroys any statistical coupling between the internal and external states but preserves the correlation structure of fluctuations within either subset. The distribution of the ensuing χ^2^ statistics (over 82 external elements) is shown in (*b*) for the true (black) and null (white) analyses. Crucially, five of the subsystems in the true analysis exceeded the largest statistic in the null analysis. The largest value of the null distribution provides protection against false positives at a level of 1/82. The probability of obtaining five χ^2^ values above this threshold by chance is vanishingly small *p* = 0.00052.

To test for statistical dependencies, the principal patterns of activity among the internal (functional) states were summarized using singular value decomposition and temporal embedding (figure 4). A classical canonical variates analysis was then used to assess the significance of a simple linear mapping between expression of these patterns and the movement of each external subsystem. Figure 4*a* illustrates these internal dynamics, while figure 4*c* shows the Newtonian motion of the external subsystem that was best predicted. The agreement between the actual (dotted line) and predicted (solid line) motion is self-evident, particularly around the negative excursion at 300 s. The internal dynamics that predict this event appear to emerge in their fluctuations before the event itself (figure 4)—as would be anticipated if internal events are modelling external events. Interestingly, the subsystem best predicted was the furthest away from the internal states (magenta circle in figure 4*d*).

This example illustrates how internal states infer or register distant events in a way that is not dissimilar to the perception of auditory events through sound waves—or the way that fish sense movement in their environment. Figure 4*d* also shows the subsystems whose motion could be predicted reliably. This predictability is the most significant at the periphery of the ensemble, where the ensemble has the greatest latitude for movement. These movements are coupled to the internal states—via the Markov blanket—through generalized synchrony. Generalized synchrony refers to the synchronization of chaotic dynamics, usually in skew-product (master-slave) systems [53,54]. However, in our set-up there is no master–slave relationship but a circular causality induced by the Markov blanket. Generalized synchrony was famously observed by Huygens in his studies of pendulum clocks—that synchronized themselves through the imperceptible motion of beams from which they were suspended [55]. This nicely illustrates the ‘action at a distance’ caused by chaotically synchronized waves of motion. Circular causality begs the question of whether internal states predict external causes of their sensory states or actively cause them through action. Exactly the same sorts of questions apply to perception [56,57]: for example, are visually evoked neuronal responses caused by external events or by our (saccadic eye) movements?

### 3.5. Autopoiesis and structural integrity

The previous section applied a simple sort of brain mapping to establish the statistical dependencies between external and internal states—and their functional correlates. The final simulations also appeal to procedures in the biological sciences—in particular neuropsychology to examine the effects of lesions. To test for autopoietic maintenance of structural and functional integrity, the sensory, active and internal subsystems were selectively lesioned—by rendering them functionally closed—in other words, by preventing them from influencing their neighbours. This is a relatively mild lesion, in the sense that they remain physically coupled with intact dynamics that respond to neighbouring elements. Because active states depend only on sensory and internal states one would expect to see a loss of structural integrity not only with lesions to action but also to sensory and internal states that are an integral part of active inference.

**Figure 5.**
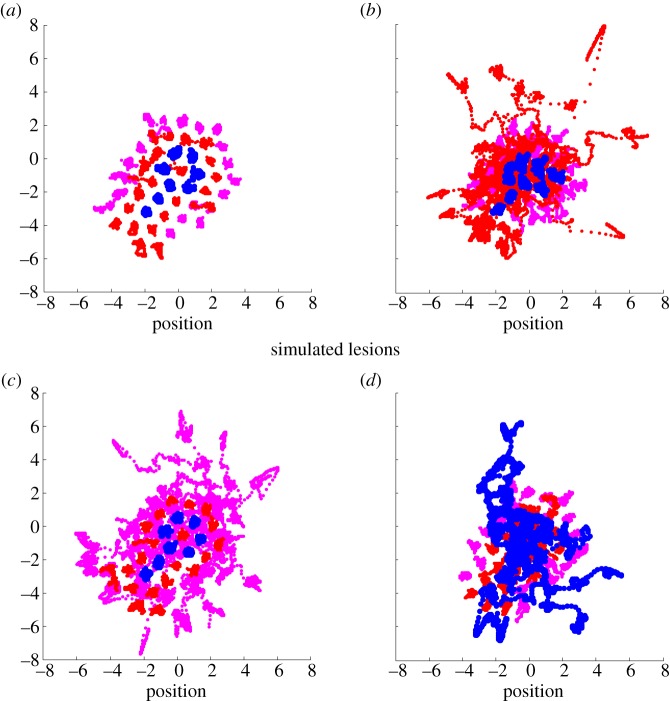
Autopoiesis and oscillator death. These results show the trajectory of the subsystems for 512 s after the last time point characterized in the previous figures. (*a*) The trajectories under the normal state of affairs; showing a preserved and quasicrystalline arrangement of the internal states (blue) and the Markov blanket (active states in red and sensory states in magenta). Contrast this formal self-organization with the decay and dispersion that ensues when the internal states and Markov blankets are synthetically lesioned (*b*,*c*,*d*). In all simulations, a subset of states was lesioned by simply rendering their subsystems closed—in other words, although the Newtonian interactions were preserved, they were unable to affect the functional states of neighbouring subsystems. (*b*) The effect of this relatively subtle lesion on active states—that are rapidly expelled from the interior of the ensemble, allowing sensory states to invade and disrupt the internal states. A similar phenomenon is seen when the sensory states were lesioned (*c*)—as they drift out into the external system. There is a catastrophic loss of structural integrity when the internal states themselves cannot affect each other, with a rapid migration of internal states through and beyond their Markov blanket (*d*). These simulations illustrate the effective death of biological self-organization that is a well-known phenomenon in dynamical systems theory—known as oscillator death: see [58]. In our setting, they are a testament to autopoiesis or self-creation—in the sense that self-organized dynamics are necessary to maintain structural or configurational integrity.

Figure 5 illustrates the effects of these interventions by following the evolution of the internal states and their Markov blanket over 512 s. Figure 5*a* shows the conservation of structural (and implicitly functional) integrity in terms of spatial configuration over time. Contrast this with the remaining three panels that show structural disintegration as the integrity of the Markov blanket is lost and internal elements are extruded into the environment.

## 4. Conclusion

Clearly, there are many issues that need to be qualified and unpacked under this formulation. Perhaps the most prescient is its focus on boundaries or Markov blankets. This contrasts with other treatments that consider the capacity of living organisms to reproduce by passing genetic material to their offspring [1]. In this context, it is not difficult to imagine extending the simulations above to include slow (e.g. diurnal) exogenous fluctuations—that cause formally similar Markov blankets to dissipate and reform in a cyclical fashion. The key question would be whether the internal states of a system in one cycle induce—or code for—the formation of a similar system in the next.

The central role of Markov blankets speak to an important question: is there a unique Markov blanket for any given system? Our simulations focused on the principal Markov blanket—as defined by spectral graph theory. However, a system can have a multitude of partitions and Markov blankets. This means that there are many partitions that—at some spatial and temporal scale—could show lifelike behaviour. For example, the Markov blanket of an animal encloses the Markov blankets of its organs, which enclose Markov blankets of cells, which enclose Markov blankets of nuclei and so on. Formally, every Markov blanket induces active (Bayesian) inference and there are probably an uncountable number of Markov blankets in the universe. Does this mean there is lifelike behaviour everywhere or is there something special about the Markov blankets of systems we consider to be alive?

Although speculative, the answer probably lies in the statistics of the Markov blanket. The Markov blanket comprises a subset of states, which have a marginal ergodic density. The entropy of this marginal density reflects the dispersion or invariance properties of the Markov blanket, suggesting that there is a unique Markov blanket that has the smallest entropy. One might conjecture that minimum entropy Markov blankets characterize biological systems. This conjecture is sensible in the sense that the physical configuration and dynamical states that constitute the Markov blanket of an organism—or organelle—change slowly in relation to the external and internal states it separates. Indeed, the physical configuration must be relatively constant to avoid destroying anti-edges (the absence of an edge or coupling) in the adjacency matrix that defines the Markov blanket. This perspective suggests that there may be ways of characterizing the statistics (e.g. entropy) of Markov blankets that may quantify how lifelike they appear. Note from equation (2.9) that systems (will appear to) place an upper bound on the entropy of the Markov blanket (and internal states). This means that the marginal ergodic entropy measures the success of this apparent endeavour.

However, minimum entropy is clearly not the whole story, in the sense that biological systems act on their environment—unlike a petrified stone with low entropy. In the language of random attractors, the (internal and Markov blanket) states of a system have an attracting set that is *space filling but has a small measure* or entropy—where the measure or volume upper bounds the entropy [11]. Put simply, biological systems move around in their state space but revisit a limited number of states. This space filling aspect of attracting sets may rest on the divergence-free or solenoidal flow (equation (2.3)) that we have largely ignored in this paper but may hold the key for characterizing life forms.

Clearly, the simulations in this paper are a long way off accounting for the emergence of biological structures such as complex cells. The examples presented above are provided as proof of principle and are as simple as possible. An interesting challenge now will be to simulate the emergence of multicellular structures using more realistic models with a greater (and empirically grounded) heterogeneity and formal structure. Having said this, there is a remarkable similarity between the structures that emerge from our simulations and the structure of viruses. Furthermore, the appearance of little cilia (figure 3) are very reminiscent of primary cilia, which typically serve as sensory organelles and play a key role in evolutionary theory [59].

A related issue is the nature of the dynamical (molecular or cellular) constituents of the ensembles considered above. Nothing in this treatment suggests a special role for carbon-based life or, more generally, the necessary conditions for life to emerge. The contribution of this work is to note that if systems are ergodic and possess a Markov blanket, they will—almost surely—show lifelike behaviour. However, this does not address the conditions that are necessary for the emergence of ergodic Markov blankets. There may be useful constraints implied by the existence of a Markov blanket (whose constituency has to change more slowly than the states of its constituents). For example, the spatial range of electrochemical forces, temperature and molecular chemistry may determine whether the physical motion of molecules (that determine the integrity of the Markov blanket) is large or small in relation to fluctuations in electrochemical states (that do not). However, these questions are beyond the scope of this paper and may be better addressed in computational chemistry and theoretical biology.

This touches on another key issue, namely that of evolution. In this treatment, we have assumed biological systems are ergodic. Clearly, this is a simplification, in that real systems are only locally ergodic. The implication here is that self-organized systems cannot endure indefinitely and are only ergodic over a particular (somatic) timescale, which raises the question of evolutionary timescales: is evolution itself the slow and delicate unwinding of a trajectory through a vast state space—as the universe settles on its global random attractor? The intimation here is that adaptation and evolution may be as inevitable as the simple sort of self-organization considered in this paper. In other words, the very existence of biological systems necessarily implies they will adapt and evolve. This is meant in the sense that any system with a random dynamical attractor will *appear to* minimize its variational free energy and can be interpreted as engaging in active inference—acting upon its external milieu to maintain an internal homoeostasis. However, the ensuing homoeostasis is as illusory as the free energy minimization upon which it rests. Does the same apply to adaptation and evolution?

Adaptation on a somatic timescale has been interpreted as optimizing the parameters of a generative model (encoded by slowly changing internal states like synaptic connection strengths in the brain) such that they minimize free energy. It is fairly easy to show that this leads to Hebbian or associative plasticity of the sort that underlies learning and memory [21]. Similarly, at even longer timescales, evolution can be cast in terms of free energy minimization—by analogy with Bayesian model selection based on variational free energy [60]. Indeed, free energy functionals have been invoked to describe natural selection [61]. However, if the minimization of free energy is just a corollary of descent onto a global random attractor, does this mean that adaptation and evolution are just ways of describing the same thing? The answer to this may not be straightforward, especially if we consider the following possibility: if self-organization has an inferential aspect, what would happen if systems believed their attracting sets had low entropy. If one pursues this in a neuroscience setting, one arrives at a compelling explanation for the way we adaptively sample our environments—to minimize uncertainty about the causes of sensory inputs [62]. In short, this paper has only considered inference as emergent property of self-organization—not the nature of implicit (prior) beliefs that underlie inference.

## References

[1] Schrödinger E 1944 What is life?: the physical aspect of the living cell. Dublin, Ireland: Trinity College.

[2] Ashby WR 1947 Principles of the self-organizing dynamic system. J. Gen. Psychol. 37, 125–128. (10.1080/00221309.1947.9918144) 20270223

[3] Haken H 1983 Synergetics: an introduction. Non-equilibrium phase transition and self-selforganisation in physics, chemistry and biology, 3rd edn Berlin, Germany: Springer.

[4] Maturana HR Varela F (eds) 1980 Autopoiesis and cognition. Dordrecht, The Netherlands: Reidel.

[5] Nicolis G Prigogine I 1977 Self-organization in non-equilibrium systems. New York, NY: Wiley.

[6] Ao P 2009 Global view of bionetwork dynamics: adaptive landscape. J. Genet. Genom. 36, 63–73. (10.1016/S1673-8527(08)60093-4) PMC3165055 19232305

[7] Demetrius L 2000 Thermodynamics and evolution. J. Theor. Biol. 206, 1–16. (10.1006/jtbi.2000.2106) 10968933

[8] Davis MJ 2006 Low-dimensional manifolds in reaction-diffusion equations. I. Fundamental aspects. J. Phys. Chem. A 110, 5235–5256. (10.1021/jp055592s) 16623450

[9] Auletta G 2010 A paradigm shift in biology? Information 1, 28–59. (10.3390/info1010028)

[10] Rabinovich MI Afraimovich VS Bick V Varona P 2012 Information flow dynamics in the brain. Phys. Life Rev. 9, 51–73. (10.1016/j.plrev.2011.11.002) 22119154

[11] Friston K 2012 A free energy principle for biological systems. Entropy 14, 2100–2121. (10.3390/e14112100) 23204829 PMC3510653

[12] Friston K Ao P 2012 Free-energy, value and attractors. Comput. Math. Meth. Med. 2012, 937860 (10.1155/2012/937860) PMC3249597 22229042

[13] Conant RC Ashby RW 1970 Every good regulator of a system must be a model of that system. Int. J. Systems Sci. 1, 89–97. (10.1080/00207727008920220)

[14] Evans DJ 2003 A non-equilibrium free energy theorem for deterministic systems. Mol. Phys. 101, 15 551–15 554. (10.1080/0026897031000085173)

[15] Evans DJ Searles DJ 1994 Equilibrium microstates which generate second law violating steady states. Phys. Rev. E 50, 1645–1648. (10.1103/PhysRevE.50.1645) 9962139

[16] Dayan P Hinton GE Neal R 1995 The Helmholtz machine. Neural Comput. 7, 889–904. (10.1162/neco.1995.7.5.889) 7584891

[17] Gregory RL 1980 Perceptions as hypotheses. Phil. Trans. R. Soc. Lond. B 290, 181–197. (10.1098/rstb.1980.0090) 6106237

[18] Helmholtz H 1866/1962 Concerning the perceptions in general. *In* Treatise on physiological optics, 3rd edn New York, NY: Dover.

[19] Kersten D Mamassian P Yuille A 2004 Object perception as Bayesian inference. Annu. Rev. Psychol. 55, 271–304. (10.1146/annurev.psych.55.090902.142005) 14744217

[20] Lee TS Mumford D 2003 Hierarchical Bayesian inference in the visual cortex. J. Opt. Soc. Am. Opt. Image Sci. Vis. 20, 1434–1448. (10.1364/JOSAA.20.001434) 12868647

[21] Friston K Kilner J Harrison L 2006 A free energy principle for the brain. J. Physiol. Paris 100, 70–87. (10.1016/j.jphysparis.2006.10.001) 17097864

[22] Ay N Bertschinger N Der R Güttler F Olbrich E 2008 Predictive information and explorative behavior of autonomous robots. Eur. Phys. J. B 63, 329–339. (10.1140/epjb/e2008-00175-0)

[23] Bialek W Nemenman I Tishby N 2001 Predictability, complexity, and learning. Neural Comput. 13, 2409–2463. (10.1162/089976601753195969) 11674845

[24] Tishby N Polani D 2010 Information theory of decisions and actions. In Perception–reason–action cycle: models, algorithms and systems (eds Cutsuridis V Hussain A Taylor J), pp. 1–37. Berlin, Germany: Springer.

[25] Soodak H Iberall A 1978 Homeokinetics: a physical science for complex systems. Science 201, 579–582. (10.1126/science.201.4356.579) 17794110

[26] Birkhoff GD 1931 Proof of the ergodic theorem. Proc. Natl Acad. Sci. USA 17, 656–660. (10.1073/pnas.17.12.656) 16577406 PMC1076138

[27] Moore CC 1966 Ergodicity of flows on homogeneous spaces. Am. J. Math. 88, 154–178. (10.2307/2373052)

[28] Pearl J 1988 Probabilistic reasoning in intelligent systems: networks of plausible inference. San Fransisco, CA: Morgan Kaufmann.

[29] Crauel H Flandoli F 1994 Attractors for random dynamical systems. Probab. Theory Relat. Fields 100, 365–393. (10.1007/BF01193705)

[30] Crauel H 1999 Global random attractors are uniquely determined by attracting deterministic compact sets. Ann. Mat. Pura Appl. 4, 57–72. (10.1007/BF02505989)

[31] Frank TD 2004 Nonlinear Fokker–Planck equations: fundamentals and applications. Springer Series in Synergetics. Berlin, Germany: Springer.

[32] Ao P 2004 Potential in stochastic differential equations: novel construction. J. Phys. A 37, L25–L30. (10.1088/0305-4470/37/3/L01)

[33] Yuan R Ma Y Yuan B Ping A 2010 Bridging engineering and physics: Lyapunov function as potential function. See http://arxiv.org/abs/1012.2721v1 [nlin.CD].

[34] Beal MJ 2003 Variational algorithms for approximate Bayesian inference. PhD thesis, University College London.

[35] Feynman RP 1972 Statistical mechanics. Reading, MA: Benjamin.

[36] Hinton GE van Camp D 1993 Keeping neural networks simple by minimizing the description length of weights. Proc. COLT-93, 5–13. (10.1145/168304.168306)

[37] Kass RE Steffey D 1989 Approximate Bayesian inference in conditionally independent hierarchical models (parametric empirical Bayes models). J. Am. Stat. Assoc. 407, 717–726. (10.1080/01621459.1989.10478825)

[38] Kullback S Leibler RA 1951 On information and sufficiency. Ann. Math. Statist. 22, 79–86. (10.1214/aoms/1177729694)

[39] van Leeuwen C 1990 Perceptual-learning systems as conservative structures: is economy an attractor? Psychol. Res. 52, 145–152. (10.1007/BF00877522) 2281126

[40] Pasquale V Massobrio P Bologna LL Chiappalone M Martinoia S 2008 Self-organization and neuronal avalanches in networks of dissociated cortical neurons. Neuroscience 153, 1354–1369. (10.1016/j.neuroscience.2008.03.050) 18448256

[41] Friston K 2010 The free-energy principle: a unified brain theory? Nat. Rev. Neurosci. 11, 127–138. (10.1038/nrn2787) 20068583

[42] Barlow H 1961 Possible principles underlying the transformations of sensory messages. In Sensory communication (ed. Rosenblith W), pp. 217–234. Cambridge, MA: MIT Press.

[43] Linsker R 1990 Perceptual neural organization: some approaches based on network models and information theory. Annu. Rev. Neurosci. 13, 257–281. (10.1146/annurev.ne.13.030190.001353) 2183677

[44] Jaynes ET 1957 Information theory and statistical mechanics. Phys. Rev. Ser. II 106, 620–630.

[45] Breakspear M Stam CJ 2005 Dynamics of a neural system with a multiscale architecture. Phil. Trans. R. Soc. B 360, 1051–1074. (10.1098/rstb.2005.1643) 16087448 PMC1854927

[46] Bressler SL Tognoli E 2006 Operational principles of neurocognitive networks. Int. J. Psychophysiol. 60, 139–148. (10.1016/j.ijpsycho.2005.12.008) 16490271

[47] Freeman WJ 1994 Characterization of state transitions in spatially distributed, chaotic, nonlinear, dynamical systems in cerebral cortex. Integr. Physiol. Behav. Sci. 29, 294–306. (10.1007/BF02691333) 7811649

[48] Turing AM 1952 The chemical basis of morphogenesis. Phil. Trans. R. Soc. Lond. B 237, 37–72. (10.1098/rstb.1952.0012) PMC4360114 25750229

[49] Belousov BP 1959 Периодически действующая реакция и ее механизм [Periodically acting reaction and its mechanism]. Сборнрефератов по радиационной медицине [Collection of Abstracts on Radiation Medicine], 145–147.

[50] Hu A Xu Z Guo L 2010 The existence of generalized synchronization of chaotic systems in complex networks. Chaos 20, 013112 (10.1063/1.3309017) 20370267

[51] Poland D 1993 Cooperative catalysis and chemical chaos: a chemical model for the Lorenz equations. Physica D 65, 86–99. (10.1016/0167-2789(93)90006-M)

[52] Zeki S 2005 The Ferrier lecture 1995 behind the seen: the functional specialization of the brain in space and time. Phil. Trans. R. Soc. Lond. B 360, 1145–1183. (10.1098/rstb.2005.1666) 16147515 PMC1609195

[53] Hunt B Ott E Yorke J 1997 Differentiable synchronisation of chaos. Phys. Rev. E 55, 4029–4034. (10.1103/PhysRevE.55.4029)

[54] Barreto E Josic K Morales CJ Sander E So P 2003 The geometry of chaos synchronization. Chaos 13, 151–164. (10.1063/1.1512927) 12675422

[55] Huygens C 1673 Horologium oscillatorium. France: Parisiis.

[56] Adams RA Shipp S Friston KJ 2012 Predictions not commands: active inference in the motor system. Brain Struct. Funct. 218, 611–643. (10.1007/s00429-012-0475-5) PMC3637647 23129312

[57] Wurtz RH McAlonan K Cavanaugh J Berman RA 2011 Thalamic pathways for active vision. Trends Cogn. Sci. 5, 177–184. (10.1016/j.tics.2011.02.004) PMC3070860 21414835

[58] De Monte S d'Ovidio F Mosekilde E 2003 Coherent regimes of globally coupled dynamical systems. Phys. Rev. Lett. 90, 054102 (10.1103/PhysRevLett.90.054102) 12633359

[59] Pallen MJ Matzke NJ 2006 From the origin of species to the origin of bacterial flagella. Nat. Rev. Microbiol. 4, 784–790. (10.1038/nrmicro1493) 16953248

[60] Friston K Penny W 2011 Post hoc Bayesian model selection. Neuroimage 56, 2089–2099. (10.1016/j.neuroimage.2011.03.062) 21459150 PMC3112494

[61] Sella G Hirsh AE 2005 The application of statistical physics to evolutionary biology. Proc. Natl Acad. Sci. USA 102, 9541–9546. (10.1073/pnas.0501865102) 15980155 PMC1172247

[62] Friston K Adams RA Perrinet L Breakspear M 2012 Perceptions as hypotheses: saccades as experiments. Front. Psychol. 3, 151 (10.3389/fpsyg.2012.00151) 22654776 PMC3361132

